# uCite: The union of nine large-scale public PubMed citation datasets with reliability filtering

**DOI:** 10.1016/j.dib.2025.111535

**Published:** 2025-04-02

**Authors:** Liri Fang, Malik Oyewale Salami, Griffin M. Weber, Vetle I. Torvik

**Affiliations:** a School of Information Sciences, University of Illinois Urbana-Champaign, Champaign, IL, United States; b Department of Biomedical Informatics, Harvard Medical School, Boston, MA, United States

**Keywords:** Citation data, Citation errors, Bibliographic database

## Abstract

There has been a recent push to make public, aggregate, and increase coverage of bibliographic citation data. Here we describe uCite, a citation dataset containing 564 million PubMed citation pairs aggregated from the following nine sources: PubMed Central, iCite, OpenCitations, Dimensions, Microsoft Academic Graph, Aminer, Semantic Scholar, Lens, and OpCitance. Of these, 51 million (9%) were labeled unreliable, as determined by patterns of source discrepancies explained by ambiguous metadata, crosswalk, and typographical errors, citing future publications, and multi-paper documents. Each source contributes to improved coverage and reliability, but varies dramatically in precision and recall, estimates of which are contrasted with the Web of Science and Scopus herein.

Specifications Table

**Table:** 

Subject	Data Science; Big Data Analytics
Specific subject area	Bibliometrics, Scientometrics, Science of Science, Citation Analysis, PubMed, Citation Integration, Reliability Filtering, Meta Data
Data format	Raw, Analyzed, Filtered
Type of data	Table, Chart, Figure
Data collection	uCite dataset is constructed in three steps: (1) a PMID crosswalk, which maps nine source identifiers to the PubMed identifier (PMID), (2) the union of citation pairs from nine sources, and (3) the citation filter by reliability (i.e., identifying reliable and unreliable citations). The data was acquired from nine open-access citation data sources. 1. PubMed Central (PMC) data is collected from PubMed Central through December 2018 via Entrez Programming Utilities (E-utilities)^1^. 2. iCite (ice) version 1, a snapshot of NIH Open Citation Collection (OCC) [1], is collected through April 2019 [2]; 3. Open Citations data is in CSV version from the November 2021 dump [3]; 4. Lens is harvested through the October 2021 version^2^; 5. Microsoft Academic Graph (mag) in the 2015 version is collected [4]; 6. Aminer citations (ami) are in the 2015 version; 7. Semantic Scholar citations are harvested in the 2019 version. 8. Dimensions citation is from published PMID pairs released by [5]; 9. Patci [6] processes OpCitance citations from PubMed Central Open Access subset reference strings extracted by [7].
Data source location	All nine source datasets are stored in the uCite repository: https://doi.org/10.13012/B2IDB-6818660_V1
Data accessibility	Repository name: uCite: The union of nine large-scale public PubMed citation datasets with reliability labels Data identification number: https://doi.org/10.13012/B2IDB-6818660_V1

1 Entrez Programming Utilities: https://www.ncbi.nlm.nih.gov/books/NBK25499/

2 The Lens, (2022). https://support.lens.org/glossary/ (accessed December 11, 2022).

## 1 Value of the Data

• uCite enhances the bibliographic database PubMed, which is widely used in academic research across bibliometrics, the science of science, data mining, text mining, natural language processing, large language models, graph mining, network analysis, etc.
• uCite is a large-scale citation dataset (nearly 513 million citation pairs, after filtering) of high quality (high coverage, precision, and recall) integrated from all the largest publicly available citation sources. uCite should improve the reliability and scalability of studies in the studies that make use of PubMed and citations, such as language models, BioLinkBert [8], and MeSHier [9].
• uCite includes several additional datasets besides reliable citation pairs: a collection of unreliable citation pairs, mappings between paper identifiers and PMIDs, a collection of duplicate PMIDs, and paper metadata. These datasets could enable further studies of data quality.

## 2 Background

Since Garfield introduced the Science Citation Index [10], Clarivate's Web of Science and Elsevier's Scopus are bibliographic data sources commonly used for citation analysis. With public domain citations exceeding one billion in 2021 [11], open-access citation data from other providers, including PubMed Central, iCite, OpenCitations, Dimensions, Microsoft Academic Graph, Aminer, Semantic Scholar, Lens, and OpCitance, have expanded citation coverage. Comparative studies confirm that different citation sources share overlapping and unique citations compared with Web of Science (WoS) or Scopus [[12], [13], [14]]. Recent studies have evaluated the accuracy of the citations across several sources, revealing unreliable citations [5,14] and missing citations [15]. Combining sources enhances the bibliometric analyses, though it requires significant effort to unify and repair data [15,16] due to varying identifiers and unreliable metadata[17]. Only 40.78% of PubMed papers (1966–2015) include a DOI, complicating citation matching [18]. To address this, we produce new broad-coverage and high-precision citation data for PubMed, named uCite. We integrate nine open-access citation sources and filter the combined data with reliability. uCite comprises a reliable set of 512,615,675 citation pairs and an unreliable set of 51,263,076 citation pairs (9.1% of all citations).

## 3 Data Description

uCite, the union of nine large-scale open-access citation data for PubMed, is freely available on Illinois Data Bank [19]. uCite contains twenty files, including the reliable and unreliable citation pairs, non-PMID identifiers to PMID mapping (for DOIs, Lens, MAG, and Semantic Scholar), original PMID pairs from the nine resources, metadata for PMIDs, duplicate PMIDs, redirected PMID pairs, and PMC OA Patci citation matching results. Table 1 describes all twenty data files. PPUB.tsv.gz and PUNR.tsv.gz are the final outputs of uCite. PPUB.tsv.gz comprises the reliable citation pairs described by PMIDs and publication years, and PUNR.tsv.gz contains the unreliable citation pairs.

**Table 1:** The description of the data that is shared in the uCite repository.

File Name	# Records	Short Description
PPUB.tsv.gz	512,615,675	tsv format file contains reliable citation pairs uCite.
PUNR.tsv.gz	51,263,076	tsv format file contains unreliable citation pairs uCite.
DOI2PMID.tsv.gz	21,464,534	tsv format file contains results mapping DOI to PMID.
LEN2PMID.tsv.gz	202,520,365	tsv format file contains results mapping LensID pairs to PMID pairs.
MAG2PMIDsorted.tsv.gz	22,290,870	tsv format file contains results mapping MAG ID to PMID.
SEM2PMID.tsv.gz	29,382,920	tsv format file contains results mapping Semantic Scholar ID to PMID.
JVNPYA.tsv.gz	29,155,266	tsv format file contains metadata of papers.
TiLTyAlJVNY.tsv.gz	29,155,266	tsv format file contains metadata of papers.
PMC-OA-patci.tsv.gz	90,221,491	tsv format file contains PMC OA subset reference strings extracted and processed by Patci.
REDIRECTS.gz	10,722,984	txt file contains unreliable PMID pairs mapped to reliable PMID pairs.
REMAP	16,102	txt file contains pairs of duplicate PubMed records (lhs PMID mapped to rhs PMID).
ami_pair.tsv.gz	92,051,710	tsv format file contains all citation pairs from Aminer (2015 version).
dim_pair.tsv.gz	508,742,049	tsv format file contains all citation pairs from Dimensions [5].
ice_pair.tsv.gz	426,803,300	tsv format file contains all citation pairs from iCite (April 2019 version, version 1).
len_pair.tsv.gz	492,744,622	tsv format file contains all citation pairs from the Lens (harvested through Oct 2021).
mag_pair.tsv.gz	293,505,595	tsv format file contains all citation pairs from Microsoft Academic Graph (2015 version).
oci_pair.tsv.gz	404,191,310	tsv format file contains all citation pairs from Open Citations (Nov. 2021 dump, csv version).
pat_pair.tsv.gz	66,468,552	tsv format file contains all citation pairs from Patci (i.e., from "PMC-OA-patci.tsv.gz").
pmc_pair.tsv.gz	141,446,706	tsv format file contains all citation pairs from PubMed Central (harvested through Dec 2018 via e-Utilities).
sem_pair.tsv.gz	206,956,956	tsv format file contains all citation pairs from Semantic Scholar (2019 version).

### 3.1 Terminology

Throughout the paper, we use the following terminology. A *citation pair* refers to a pair of PMIDs (fromPMID, toPMID), where fromPMID denotes the *citing paper*, toPMID denotes the *cited paper*, and *PMID* stands for PubMed Identifier. A *solo citation* pair refers to a citation pair unique to one source, where the s*ource* refers to one of the nine public citation datasets. A *reference string* in a citing paper refers to a row in a list of references that typically appears at the end of a paper. The *reference string* does not refer to the in-text citation contexts [7]. A *record* refers to a row in a dataset. A *paper record* typically points to one paper but can point to a wrong paper (i.e., *unreliable mapping*), or multiple *paper records* can point to one paper (i.e., *duplicate records*). *Unreliable mapping* refers to the incorrect mapping from a reference string or paper metadata to a PMID. *Duplicate records* refer to cases where multiple *paper records* within a data source (e.g., multiple PMIDs or multiple DOIs) are mapped to the same paper. It should also be noted that a paper can have multiple versions, as is common for particular publication venues such as Cochrane Reviews, where PMIDs also have versions but DOIs do not. We call a paper *in-scope* (*out-of-scope*) if it is in PubMed (not in PubMed, respectively). We might also say that a paper is *out-of-scope* for a particular source, such as PMC, when the paper is in PubMed but not PMC.

The column names and descriptions of each file above are listed as follows.

**FILENAME** : *PPUB.tsv.gz, PUNR.tsv.gz*
  (1) fromPMID - PubMed ID of the citing paper.
  (2) toPMID - PubMed ID of the cited paper.
  (3) sources - citation sources, in which the citation pairs are identified.
  (4) fromYEAR - Publication year of the citing paper.
  (5) toYEAR - Publication year of the cited paper.
**FILENAME** : *DOI2PMID.tsv.gz*
  (1) DOI - Semantic Scholar ID of paper records.+
  (2) PMID - PubMed ID of paper records.
  (3) PMID2 - Digital Object Identifier of paper records, “-” if the paper doesn't have DOIs.
**FILENAME** : *SEMID2PMID.tsv.gz*
  (1) SemID - Semantic Scholar ID of paper records.
  (2) PMID - PubMed ID of paper records.
  (3) DOI - Digital Object Identifier of paper records, “-” if the paper doesn't have DOIs.
**FILENAME** : *JVNPYA.tsv.gz*
  - Each row refers to a paper record.
  (1) PMID - PubMed ID.
  (2) journal - Journal name.
  (3) volume - Journal volume.
  (4) issue - Journal issue.
  (5) pages - The paper's first and last page (without leading digits) number is separated by '-'.
  (6) year - Publication year.
  (7) lastname - Last name of the first author.
**FILENAME** : *TiLTyAlJVNY.tsv.gz*
  (1) PMID - PubMed ID.
  (2) title_tokenized - Paper title after tokenization.
  (3) languages - Language that the paper is written in.
  (4) pub_types - Types of the publication.
  (5) length(authors) - String length of author names.
  (6) journal -Journal name.
  (7) volume - Journal volume.
  (8) issue - Journal issue.
  (9) year - Publication year of print (not necessarily epub).
**FILENAME** : *PMC-OA-patci.tsv.gz*
  (1) pmcid - PubMed Central identifier.
  (2) pos -
  (3) fromPMID - PubMed ID of the citing paper.
  (4) toPMID - PubMed ID of the cited paper.
  (5) SRC - citation sources identified by the citation pairs.
  (6) MatchDB - PubMed, ADS, DBLP.
  (7) Probability - Matching probability predicted by Patci.
  (8) toPMID2 - PubMed ID of the cited paper, extracted from OA XML file
  (9) SRC2 - citation sources, in which the citation pairs are identified.
  (10) intxt_id -
  (11) journal - First character of the journal name.
  (12) same_ref_string - Y if patci and XML reference string match; otherwise, N.
  (13) DIFF -
  (14) bestSRC - Citation sources, in which the citation pairs are identified.
  (15) Match - Matching strings annotated by Patci.
**FILENAME**: *REDIRECTS.gz*
  Each row in Redirectis.txt is a string sequence in the same format.
  - “REDIRECTED FROM: source PMID_i PMID_j -> PMID_i' PMID_j”
  - “REDIRECTED TO: source PMID_i PMID_j -> PMID_i PMID_j'”
  Note: source is the name of sources where the PMID_i and PMID_j are from.
**FILENAME** : *REMAP*
  Each row is remapping unreliable PMID pairs mapped to reliable PMID pairs.
  The format of each row is “$REMAP{PMID_i} = PMID_j”.
**FILENAME** : *ami_pair.tsv.gz, dim_pair.tsv.gz, ice_pair.tsv.gz, len_pair.tsv.gz, mag_pair.tsv.gz, oci_pair.tsv.gz, pat_pair.tsv.gz, pmc_pair.tsv.gz, sem_pair.tsv.gz*
  (1) fromPMID - PubMed ID of the citing paper.
  (2) toPMID - PubMed ID of the cited paper.

### 3.2 Data evaluation

We evaluate the data quality of individual sources using six evaluation metrics: availability, coverage, scopes, completeness, precision, and recall.

Before introducing the evaluation metrics, we note that a citing paper can (and often will) cite papers that are not indexed in PubMed (i.e., some cited papers are out-of-scope). For example, in the PMC Open Access dataset, 85-95% of cited papers are indexed in PubMed, as shown in Fig. 2. The lists of cited papers are often incomplete for older citing papers in PMC because, e.g., reference strings were apparently extracted from portable document format files (PDFs), sometimes using optical character recognition technology. Out-of-scope cited papers are often conference abstracts, books, and journals not covered by PubMed. Around 1-2% of out-of-scope cited papers without PMIDs can be found in the Astrophysics Data System (ADS) or the DBLP computer science bibliography, containing papers in physics, astronomy, and computing. The following evaluation assumes that PubMed is the universe, i.e., it completely ignores cited papers that do not map to a PMID. In addition, uCite does not contain citations solely available from WoS or Scopus, as the corresponding data licenses do not permit sharing.

**Fig. 1:**
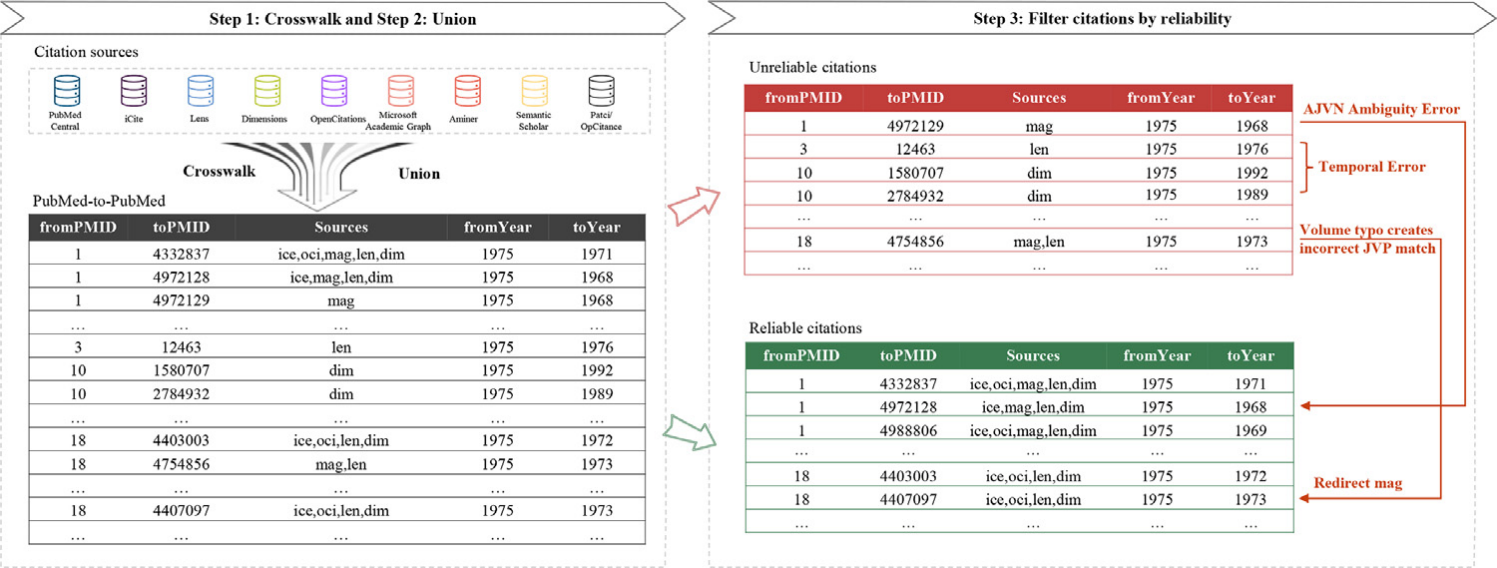
Overview of data processing with illustrative examples of data records. Step 1 is a PMID crosswalk, which refers to a mapping from source identifier to PubMed Identifier (PMID), e.g., DOI to PMID. Step 2 is a union of PMID pairs across all nine sources. Step 3 is to filter all PMID pairs by reliability. AJVN Ambiguity Error refers to a case where the author name, journal name, volume, and issue number are shared by different articles. Volume typo refers to a case where the authors wrote the wrong volume (21 instead of 22), which creates a JVP (journal, volume, and page) match to the wrong article for MAG (mag) and Lens (len). len captures both reliable and unreliable citation pairs. mag only captures the unreliable one and is redirected.

**Fig. 2:**
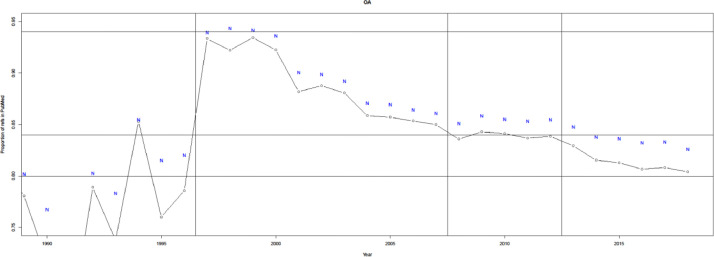
Completeness of cited papers based on the PMC OA subset. Black circles denote PubMed only, and Blue N includes (1-2\%) cited papers to ADS or DBLP as well.

The overview of evaluation statistics is listed in Table 2, including availability, coverage, completeness, precision, and recall. uCite stands out with the highest availability, containing 91.7% of papers in 2017 with at least one cited paper, closely followed by Dimensions (dim) and Lens (len). It is important to note that among the data sources that constitute uCite, no single source excels in all evaluation metrics. Lens (len) achieves the highest overall coverage score (94.4% after 1995) compared to other data sources. Patci/OpCitance (pat) and PMC (pmc) reach the top category of precision scores at 99.97% and 99.96%. However, PMC's overall coverage score is relatively lower at 30.1%. Additionally, Patci/OpCitance achieves the highest recall score (99.9%), although its availability score is not exceptionally high (25.7%). Therefore, uCite unites and filters all nine data sources to effectively combine the data sources and filter the unreliable citations.

**Table 2:** Evaluation statistics on each data source. (OA) represents the recall score compared to the PMC OA subset. The highest scores are highlighted in bold. The second-highest scores are underscored. uCite is the upper baseline to measure completeness, precision, and recall. Asteroid (*) highlights that the coverage scores of uCite, sco, and wos are based on all of the citation pairs and differ from other sources based on the uCite.

Data Source	Availability (citation)			Overall Coverage		Unique coverage			Completeness		Precision	Recall of extraction and match			
												Public		OA	
	1990	2010	2017	pre. 1995	after 1995	pre. 1995	1995-2008	after 2008	pre. 1995	after 1995		pre. 1995	after 1995	pre. 1995	after 1995
uCite	**54.2**	**86.9**	**91.7**	76.5*	93.8*	-	-	-	-	-	-	-	-	-	-
len	48.0	82.6	87.9	**89.8**	**94.4**	**46.4**	**35.9**	**35.8**	91.8	97.8	98.7	92.8	98.1	99.0	**99.9**
dim	48.1	80.1	89.9	85.0	93.0	37.7	32.5	28.3	90.6	97.3	99.6	90.7	97.4	99.4	**99.9**
ice	40.8	74.4	85.1	71.3	85.1	1.53	1.82		89.9	95.7	99.8	90.3	95.8	98.9	99.8
oci	39.7	73.2	82	61.6	78.0	4.40	9.33		79.1	90.6	99.7	88.7	94.4	90.9	94.7
mag	31.2	69.5	0.17	46.0	70.6	1.87	2.97		71.6	84.7	87	75.6	86.6	75.8	89.2
sem	8.16	46.5	53.1	11.6	46.1	7.93	13.7	26.8	69.8	88.7	97.3	74.0	90	81.6	93.1
pmc	6.12	22.8	36.2	13.5	30.1	-	-	-	94.4	99.1	99.96	95.2	99.2	98.8	99.7
ami	14.9	44.0	0.06	10.1	25.9	-	-	-	-	-	95.7	-	72.8	-	78.2
pat	0.07	8.75	25.7	0.07	14.6	-	-	-	**99.8**	**99.9**	99.97	**99.9**	**99.9**	**99.9**	**99.9**
sco	-	-	-	-	89.4*	-	-	-	94.4	96.2	**99.97**	95.3	97.5	-	98.0
wos	-	-	-	80.3*	76.5*	-	-	-	90.3	89.2	**99.97**	96.0	96.2	95.0	97.0

### 3.3 Availability, coverage, and scope

**Availability.** Two measures of source *availability are calculated*: the proportion of total citation pairs that each source contains (Eq. (1)) and the proportion of papers with at least one cited paper (Eq. (2)).

(1) $$Availability_{\_}Citations_{s}=\frac{\sum_{\left(p_{i},p_{j}\right)}\left[\left(p_{i},p_{j}\right)\in C_{s}\right]}{\sum_{\left(p_{i},p_{j}\right)}\left[\left(p_{i},p_{j}\right)\in \cup_{s\in S}C_{s}\right]}$$

(2) $$Availability_{\_}Papers_{s}=\frac{\sum_{p_{i}\in P_{s}}\left[\sum_{p_{j}}\left(p_{i},p_{j}\right)\in C_{s}\geq 1\right]}{\sum_{p_{k}}\left[p_{k}\in \cup_{s\in S}P_{s}\right]}$$

where $\left(p_{i},p_{j}\right)\in C_{s}$ denotes a citation pair, paper $p_{i}$ citing paper $p_{j}$, from a source s. $C_{s}$ represents the set of citation pairs from a source s, where s∈S. S represents the set of all nine citation source S={PubMedCentral,iCite,OpenCitations,Dimensions,MicrosoftAcademicGraph,Aminer,SemanticScholar,Lens,Patci/OpCitance}. $\;P_{s}$ represents the papers from a source s, where s∈S.

**Fig. 3:**
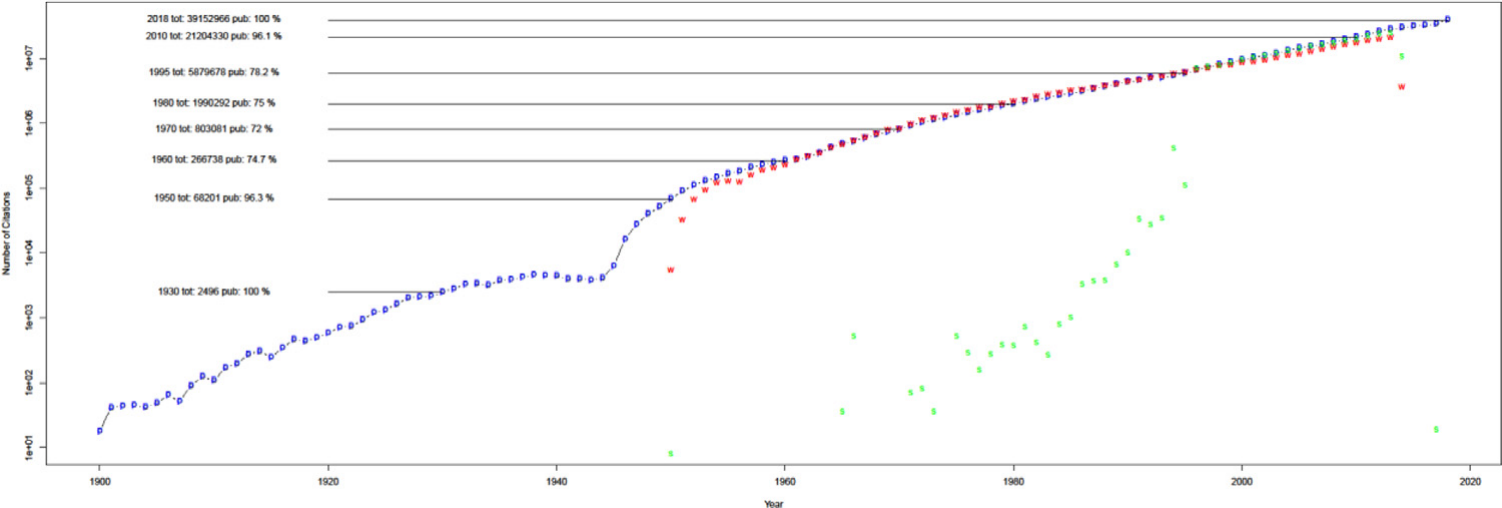
Availability of citations over time. Initial number for public uCite (p, blue) vs. wos (w, red) and sco (s, green). The black circle is the union of uCite, Scopus, and WoS. The citation availability of uCite (p, blue) is slightly higher than the union of uCite, Scopus, and WoS (circle, black). "tot: <number>" denotes the total number of citations, and "pub: <percentage>" denotes the percentage of citation availability of all public data from nine resources.

**Fig. 4:**
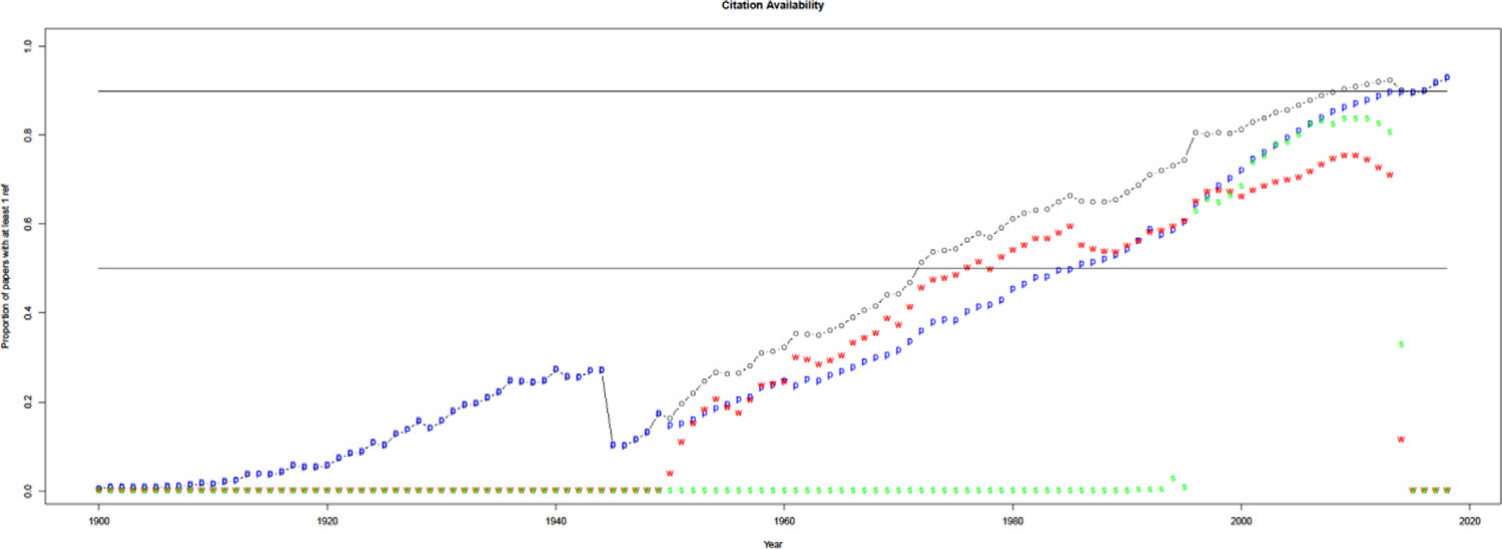
Availability of citations. The proportion of papers in PubMed with at least one ref. uCite (p, blue) vs. WoS (w, red) and Scopus (s, green). The black circle is the union of uCite, Scopus, and WoS.

**Fig. 5:**
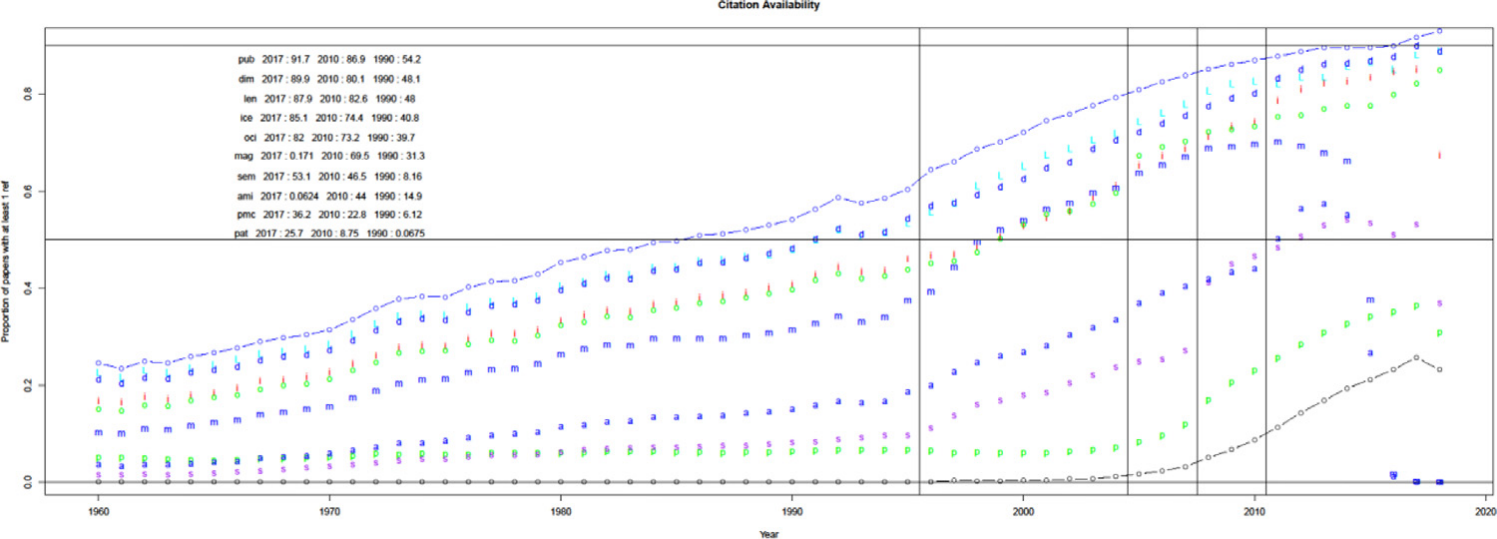
Availability of individual public sources. The sources include: union of all public (circle, blue), Dimensions (d, blue), Lens (L, light blue), iCite (i, red), OpenCitations (o, green), Microsoft Academic Graph (m, blue), Semantic Scholar (s, purple), Aminer (a, blue), PMC (p, blue), and Patci/OpCitance (circle, black).

When compared with WoS and Scopus in Fig. 3, uCite factors in an increasing proportion of papers from the union of the public dataset, Scopus, and WoS since 1970. Furthermore, the percentage of papers in uCite with at least one cited paper has increased since 1945, and availability has improved since Scopus became involved, as shown in Fig. 4. In Fig. 5, uCite achieves the highest availability of papers with at least one cited paper over time compared to nine individual citation sources. While Dimensions reaches the second highest availability of papers, it does not capture as many citation pairs as other sources and has lower precision than, for example, pmc, ice, and oci.

**Coverage.** Coverage is the proportion of citation pairs in the public data (i.e., uCite) that a data source contains. We evaluate the coverage of each data source with overall coverage by source (Eq. (3)) and unique coverage by source (Eq. (4)). The overall coverage by source represents the portion of citation pairs in uCite contained by at least one source. The unique coverage by source is the proportion of citation pairs contained by only one source. Moreover, we compare the coverage of each public data source with citation data from Web of Science and Scopus.

(3) $$Coverage\_Overall_{s}=\frac{\sum_{\left(p_{i},p_{j}\right)}\left[\left(p_{i},p_{j}\right)\in C_{s}\cap C_{\text{uCite}}\right]}{\sum_{\left({p_{i}}^{\prime },{p_{j}}^{\prime }\right)}\left[\left({p_{i}}^{\prime },{p_{j}}^{\prime }\right)\in C_{\text{uCite}}\right]}$$

(4) $$Coverage\_Unique_{s}=\frac{\sum_{\left(p_{i},p_{j}\right)}\left[\left(p_{i},p_{j}\right)\in C_{s}\cap C_{\text{uCite}}\right]\left[\left(p_{i},p_{j}\right)\notin \cup_{k\in \left\{S\setminus s\right\}}C_{k}\right]}{\sum_{s^{\prime }\in S}\sum_{\left(p_{i},p_{j}\right)}\left[\left(p_{i},p_{j}\right)\in C_{s^{\prime }}\cap C_{\text{uCite}}\right]\left[\left(p_{i},p_{j}\right)\notin \cup_{k^{\prime }\in \left\{S\setminus s^{\prime }\right\}}C_{k^{\prime }}\right]}$$

where $\left(p_{i},p_{j}\right)\in C_{s}$ denotes a citation pair, paper $p_{i}$ citing paper $p_{j}$. $C_{s}$ represents the set of citation pairs from a source s, where s∈S. {S∖s} represents the set of all citation sources S excluding the source s.

**Fig. 6:**
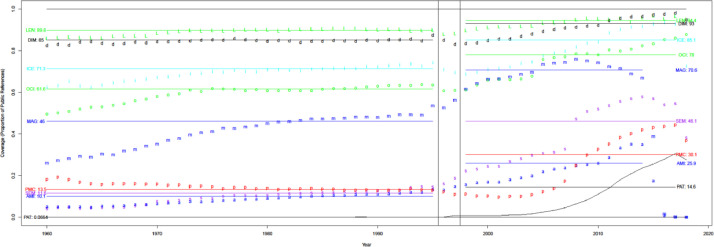
Coverage by source over time.

**Fig. 7:**
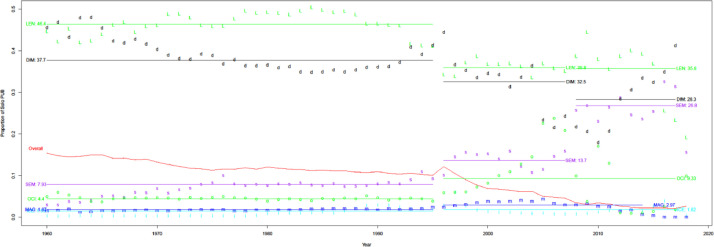
The proportion of unique coverage over the total number of solo citation pairs by source over time. The proportion of solo citation pairs covered by a source. Overall (red) denotes the overall proportion of citation pairs that are contributed by one source.

**Fig. 8:**
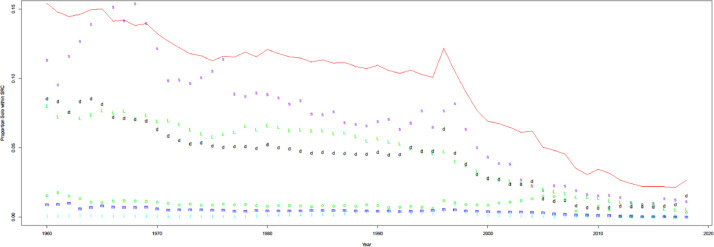
The proportion of public data only available from one source. The figure contains overall (line, red), Semantic Scholar(s, purple), Lens (L, green), Dimensions (d, black), OpenCitations (o, green), Microsoft Academic Graph (m, blue), and iCite (i, light blue).

**Fig. 9:**
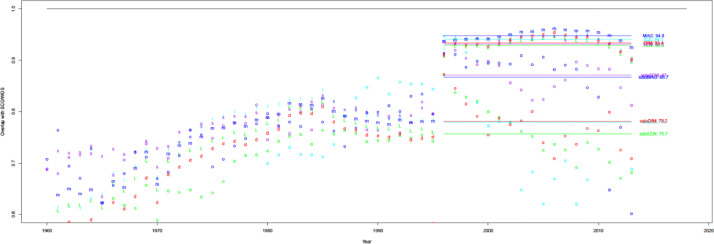
Overlap of solo public sources with wos and sco.

Fig. 6 shows the proportion of public citation pairs (i.e., uCite) each source contains. Lens(len) achieves the highest coverage proportion (94.4%) of uCite, similar to the availability results in Fig. 5. However, even Lens fails to capture more than 5% of the cited papers in uCite. Fig. 7 shows that Lens (LEN) is the source of citation pairs found by one source (35.8%) most often, but other sources also contribute. In recent years, Semantic Scholar has had a significant portion (26.8%) of unique coverage since its source is unique. Fig. 8 indicates that multiple sources cover more recent data, whereas less than 5% of public data in uCite is solo citations, covered by sole sources in recent years. This redundancy across sources can benefit the production of more reliable data. Furthermore, Fig. 9 shows a high overlap (80-90%) of solo citations with Scopus and WoS, which is high-precision non-public data. That is, the solo citation does not necessarily indicate unreliable citations but serves as independent confirmation in most cases when confirming the reliability of citation pairs.

**Scope.***in-scope of a source* denotes a paper with PMID in the scope of a source. The *out-of-scope of a source* indicates a paper with PMID that is out-of-scope of a source, such as PMC, when the paper is in PubMed but not in PMC. Eqs. (5) and (6) define functions in−scope(·,·) and out−of−scope(·,·). We call a paper *in-scope* (*out-of-scope*) if it is in PubMed (not in PubMed, respectively).

(5) $$in-scope\left(p,s\right)=\left[p\in P_{s}\right]$$

(6) $$out-of-scope\left(p,s\right)=\left[p\notin P_{s}\right]$$

(7) $$out-of-scope\;proportion_{\left(reference,\;s\right)}=\frac{\sum_{\left(p_{i},p_{j}\right)}\left[\left(p_{i},p_{j}\right)\in C_{s}\right]in-scope\left(p_{i},s\right)out-of-scope\left(p_{j},s\right)}{\sum_{\left(p_{i}^{\prime },p_{j}^{\prime }\right)}\left[\left(p_{i}^{\prime },p_{j}^{\prime }\right)\in C_{uCite\;\cup \;Scopus\;\cup \;WoS}\right]}$$

where $\left(p_{i},p_{j}\right)\in C_{s}$ denotes a citation pair, paper $p_{i}$ citing paper $p_{j}$. $C_{s}$ represents the set of citation pairs from a source s, where s∈S. {S∖s} represents the set of all citation sources S excluding the source s.

**Fig. 10:**
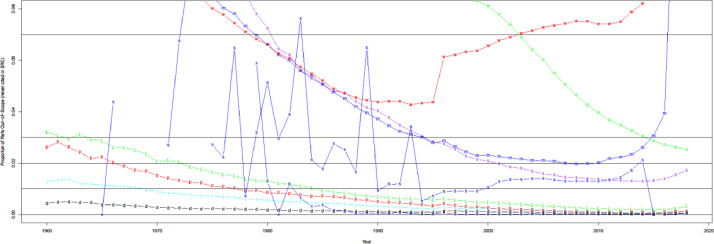
Out-of-scope references by sources. Open citations (o, green), decrease since more and more papers are getting DOIs. WoS (w, red): we include a certain version of WoS, limited to Medline (w/ MesH), not PubMed. The proportion of MEDLINE is increasing. Other sources include Scopus (s, blue), Microsoft Academic Graph (m, blue), Semantic Scholar (s, purple), Lens (L, green), PMC (p, red), Patci/OpCitance (x, blue), iCite (i, light blue), Dimensions (d, black).

As shown in Fig. 10, most sources contain less than 2% of citation pairs that are out-of-scope. In theory, citation pairs in PMC and iCite are in-scope since they use PMIDs as indices. However, pre-2000, out-of-scope estimates may be overestimated because iCite and PMC should contain all citation pairs as in-scope.

### 3.4 Accuracy

Accuracy is evaluated by three metrics: completeness, precision, and recall. Each measures a different range of citations covered by each source. Table 3 provides a summary of the three metrics' scopes. Coverage includes citing and cited papers that are out-of-scope records. Completeness contains the in-scope citing papers and cited papers that are out-of-scope records. Recall measures only in-scope citing and cited papers. Consequently, the value of coverage is smaller than recall. For example, OCIs exclude articles without DOIs since OCIs are based on DOIs. Therefore, the coverage of OCIs is expected to be smaller than recall, particularly for older articles that are less likely to have DOIs, while most recent articles have DOIs. It is worth noting that OCIs rely on Crossref as their data source, while iCite uses a subset of PMC.)

**Table 3:** Scope of evaluation metrics. “Yes” indicates the metric is restricted to the papers that are in-scope of a source. “No” includes papers that are out-of-scope of a source but in-scope of PubMed. “No*” includes papers that are out-of-scope of PubMed.

Evaluation Metrics	Has in-scope restriction?	
	Citing Paper	Cited Paper
*Availability*	No*	No*
*Coverage*	No	No
*Out-of-scope*	Yes	No
*Completeness*	Yes	No
*Recall*	Yes	Yes
*Precision*	Yes	No

***Completeness.****Completeness* is the proportion of citation pairs in the public data (i.e., uCite) that each source contains, and the citing paper is in-scope of the source.

(8) $$Completeness_{s}=\frac{\sum_{\left(p_{i},p_{j}\right)}\left[\left(p_{i},p_{j}\right)\in C_{s}\cap C_{\text{PubMed}}\right]in-scope\left(p_{i},\;s\right)}{\sum_{\left({p_{i}}^{\prime },{p_{j}}^{\prime }\right)}\left[\left({p_{i}}^{\prime },{p_{j}}^{\prime }\right)\in C_{\text{PubMed}}\right]in-scope\left({p_{i}}^{\prime },\;s\right)}$$

where $\left(p_{i},p_{j}\right)\in C_{s}$ denotes a citation pair, paper $p_{i}$ citing paper $p_{j}$. $C_{s}$ represents the set of citation pairs from a source s, where s∈S. $C_{\text{PubMed}}$ represents the set of citation pairs from PubMed. in-scope($p_{i}$, s) denotes the citing paper $p_{i}$ is in-scope of source s.

**Fig. 11:**
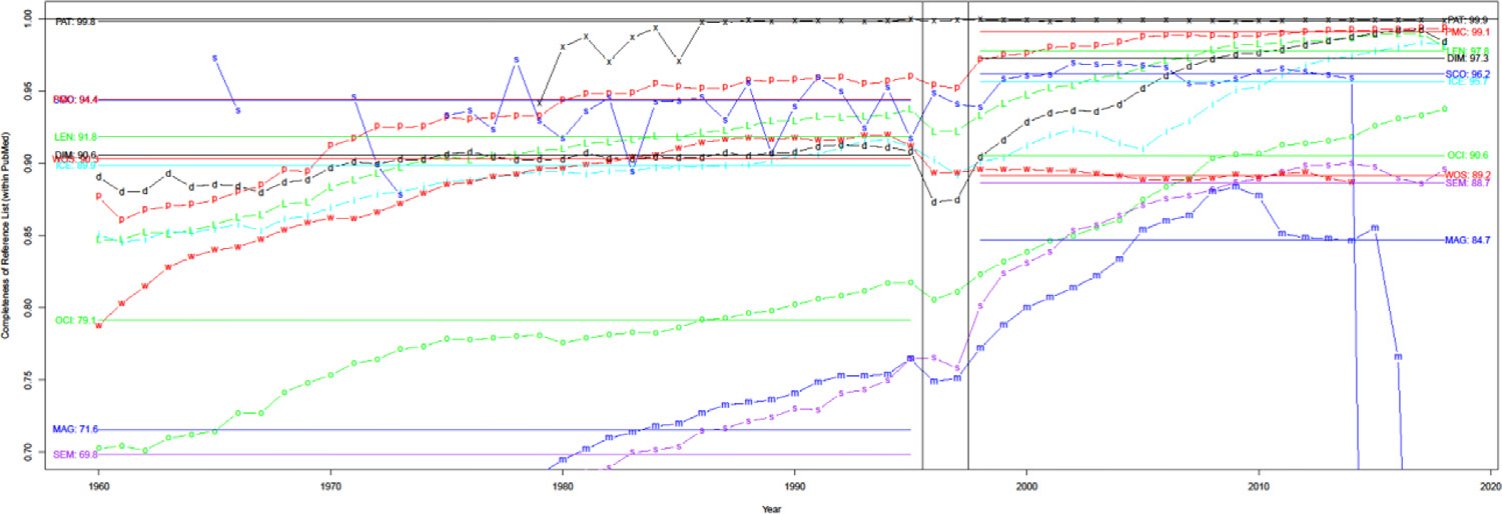
Completeness of sources relative to all PMIDs found. Black x denotes pat. The black x denotes Patci (which can be incomplete when considering WoS and Scopus).

A list of cited papers for a given source achieves 100% completeness if that source finds all cited papers within PubMed (i.e., no other source finds additional ones deemed reliable). As shown in Fig. 11, Patci/OpCitance receives the highest completeness score (99.9%) by utilizing reference strings extracted from PubMed Central XML files. uCite has reached competing completeness compared with Patci/OpCitance in recent years. However, some sources, such as WoS (89.2%) and OpenCitations (90.6%), have lower completeness partly because a large portion of PMIDs are out-of-scope.

***Precision.****Precision* measures the proportion of citation pairs correctly retrieved by each data source and implies the strictness of the matching procedure. The false positives are identified as unreliable citation pairs.

(9) $$Precision_{s}=\frac{\text{True}\;\text{Positiv}e_{s}}{\text{True}\;\text{Positiv}e_{s}+\text{False}\;\text{Positiv}e_{s}}=\frac{\sum_{\left(p_{i},p_{j}\right)}\left[\left(p_{i},p_{j}\right)\in C_{s}\cap C_{\text{uCite}}\right]}{\sum_{\left(p_{i^{\prime }},p_{j^{\prime }}\right)}\left[\left(p_{i^{\prime }},p_{j^{\prime }}\right)\in C_{s}\right]}$$

where $\left(p_{i},p_{j}\right)\in C_{s}$ denotes a citation pair of paper $p_{i}$ and paper $p_{j}$ is included in a set of citation pairs $C_{s}$. $C_{s}$ represents the set of citation pairs from a source s, where s∈S.

**Fig. 12:**
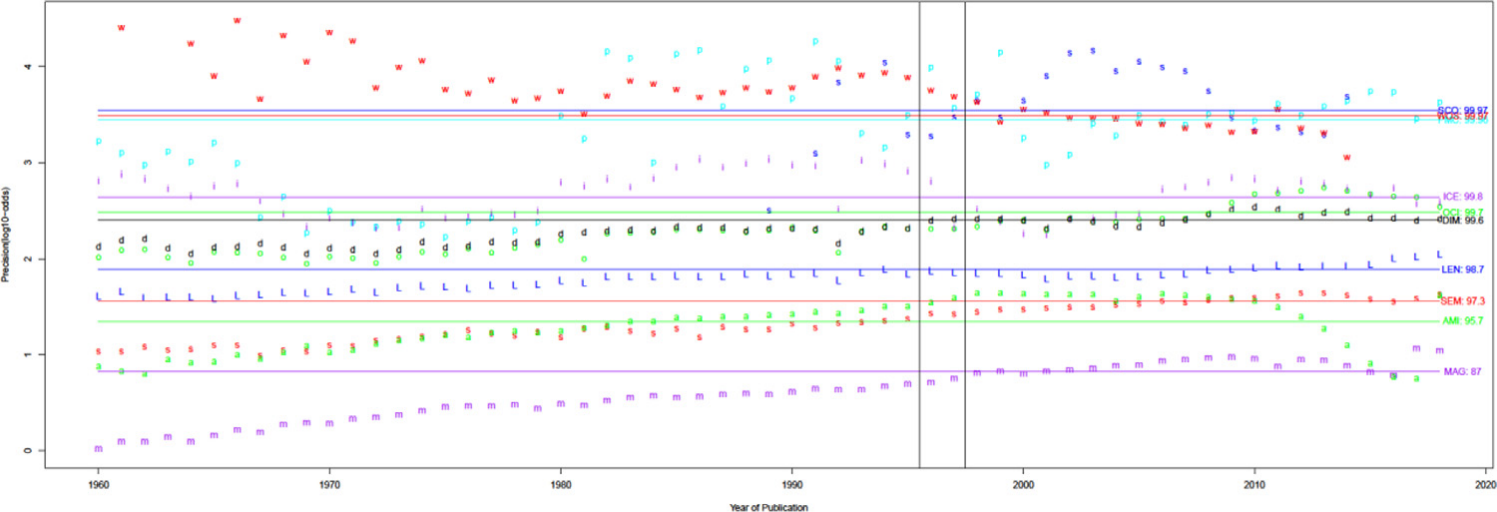
Precision over time for nine different sources. Note the log scale on the y-axis.

Fig. 12 shows that precision typically increases over time, except when earlier numbers lead to overestimation. Precision in recent years can be split into four categories among the nine citation sources. Category I contains Patci/OpCitance, PubMed Central, Scopus, and WoS, of which one unreliable citation is identified per 10k citations. Category II comprises iCite, OpenCitations, and Dimensions, with one unreliable citation per 1k citations. Category III contains Lens, Semantic Scholar, and Aminer; per 100 citations, it is considered unreliable. Finally, category IV has MAG, in which one unreliable citation is identified per 10 citations.

***Recall.****Recall* of extraction and matching procedure measures the proportion of citation pairs in uCite, of which both citing and cited papers are in the scope of a source. Recall measures whether the source should have captured the PMIDs, which implies how good the matching procedure and data quality are by source and how the in-scope papers vary widely across sources. The in-scope paper of a source denotes that the paper is identified by a PMID and is contained in the source as defined in Eqs. (5) and (6).

(10) $$Recall_{\left(\text{in}-\text{scope},\;s\right)}=\frac{\sum_{\left(p_{i},p_{j}\right)}\left[\left(p_{i},p_{j}\right)\in C_{s}\cap C_{\text{uCite}}\right]in-scope\left(p_{i},\;s\right)in-scope\left(p_{j},s\right)}{\sum_{\left({p_{i}}^{{}^{\prime }},p_{j^{\prime }}\right)}\left[\left(p_{i^{\prime }},p_{j^{\prime }}\right)\in C_{\text{PubMed}}\right]in-scope\left(p_{i^{\prime }},\;s\right)in-scope\left(p_{j^{\prime }},s\right)}$$

where $\left(p_{i},p_{j}\right)\in C_{s}$ denotes a citation pair of paper $p_{i}$ and paper $p_{j}$ is included in a set of citation pairs $C_{s}$. $C_{s}$ represents the set of citation pairs from a source s, where s∈S.

**Fig. 13:**
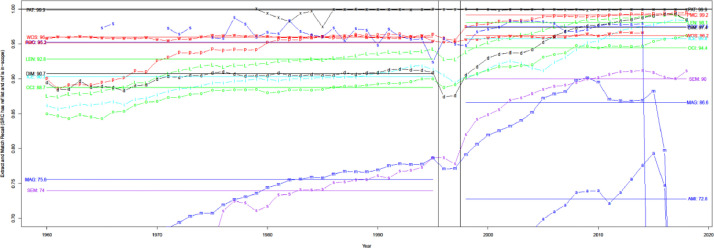
Recall of matching process (extracting reference string and matching it with an in-scope paper with PMID).

**Fig. 14:**
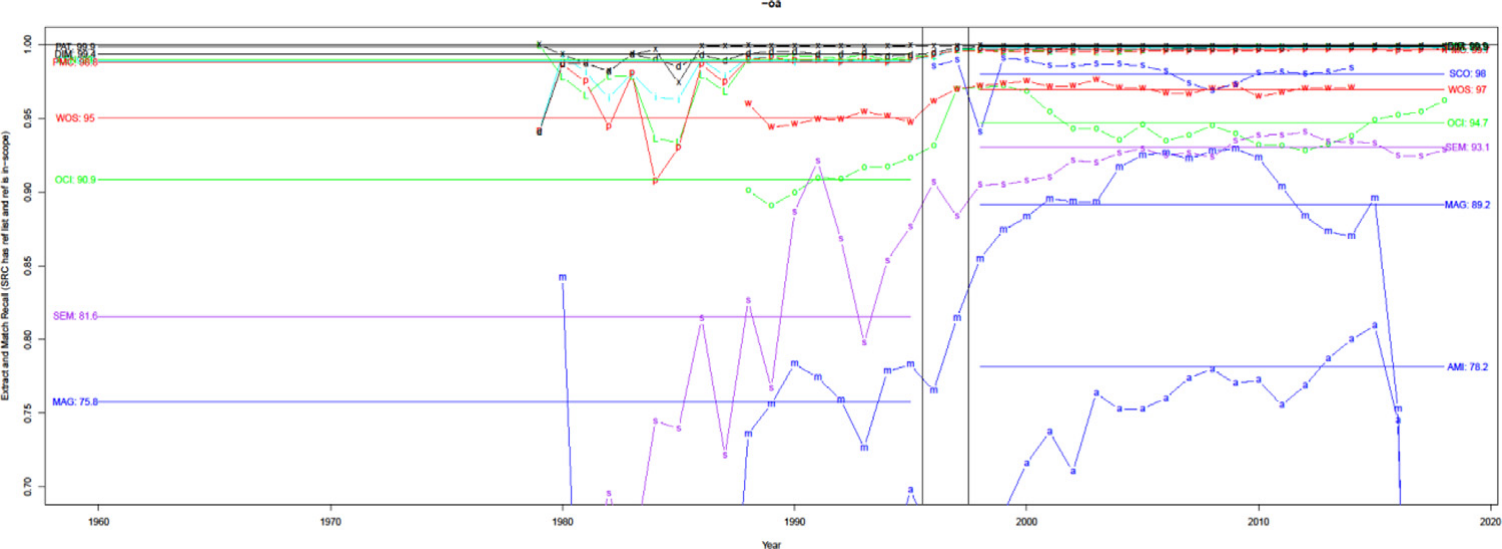
Recall of matching process for PMC OA subset.

Fig. 13 measures whether the source should have caught the PMID, as defined in Eq. (10). WoS is the only source with consistent performance from 1960-2014 but fails to capture about 3-4%. This also reflects each source operating with data of different quality, some of which are more difficult to process accurately (e.g., extracting records from old PDF and XML files). For this reason, we also consider a recall for a subset of articles shared by all the sources, i.e., the PMC Open Access subset, which gives a level playing field in Fig. 14. WoS performs slightly better for the PMC OA subset than for records with PMID but still fails to capture a significant portion of citation pairs, whereas PMC, iCite, Lens, and Patci/OpCitance capture nearly all citation pairs.

## 4 Experimental Design, Materials and Methods

Metadata inconsistencies pose a challenge when integrating different citation sources. Studies highlight issues with Digital Object Identifiers (DOIs) in WoS, Scopus, and Crossref, noting that incorrect or missing DOIs can lead to citation mismatches and inaccurate metrics [18,20]. Furthermore, other metadata fields, such as publication date and year, may contain errors and inconsistencies [17]. To address the challenge, the uCite dataset is constructed in three steps: (1) a PMID crosswalk, which maps source identifiers to PubMed ID (PMID) considering both identifiers and metadata; (2) a union of the PMID-to-PMID pairs from nine sources; and (3) the citation filter by reliability. The overview of the data processing to produce uCite is shown in Fig. 1.

### 4.1 Crosswalk and union

uCite integrates citation pairs from nine citation sources: PubMed Central, iCite, OpenCitations, Dimensions, Microsoft Academic Graph, Aminer, Semantic Scholar, Lens, and Patci/OpCitance. The details of the data version can be found in the Specifications Table. In addition, citation data from the Web of Science (WoS) and Scopus in the 2015 version are harvested as additional evidence sources to confirm the reliability of citation pairs. PMC Open Access Subset extracted by [7] is used to enrich the collection of PMID records.

Citation pairs from sources that contain identifiers (e.g., DOIs) are mapped to PMIDs. Those from sources not containing PMIDs or DOIs are mapped to PMIDs by a probabilistic matching method. Citation pairs from Lens, OpenCitations, Microsoft Academic Graph, and Semantic Scholar do not have PMIDs attached. Therefore, a crosswalk is applied to map citation pairs from the source identifier to PMID. For example, OpenCitations takes Crossref as one of the supplier database sources, and its data contains the DOIs from Crossref as one of the identifiers. Thus, DOIs are matched to the PMIDs for papers from OpenCitations. However, some papers without DOIs, such as old conference papers, might not be included in the crosswalk. For Semantic Scholar, we process DOI to PMID mapping and Semantic Scholar's mapping to PMID. For Microsoft Academic Graph, Patci is used to crosswalk between PMIDs and magIDs. lensID pairs are mapped to PMID pairs for citations from the Lens. After the crosswalk, a union of PMID pairs across all nine sources is generated and stored in a union table, as shown in the left panel of Fig. 1. The union table contains PMID pairs, sources identifying the pairs, and citing and cited years.

### 4.2 Filtering citations by reliability

The citation pairs in the union table are filtered by reliability and divided into unreliable and reliable citation sets. The nine citation sources, plus WoS and Scopus, are considered evidence sources to determine the reliability. When multiple sources identify citation pairs, they are identified as unreliable only if all these sources recognize them as unreliable. When citation pairs are only held by one particular source, multiple rules are checked to confirm the reliability of the pairs. 1) Patci/OpCitance is used to identify errors and unreliability since OpCitance provides further information on reference strings extracted from the XML file. Patci inputs the OpCitance data and predicts the probability of papers' cited papers. 2) The temporality of the citation pairs is investigated. The citation pair will be confirmed as unreliable if the citing paper is published after the cited publication. 3) Duplicated paper records are deduplicated and linked to only one PMID. 4) Unreliable citation pairs are confirmed by sources or redirected to reliable PMIDs.

### 4.3 Systematic patterns of unreliable citations

Systematic patterns of unreliable citations are used to identify unreliable citations from PMID citation pairs. These unreliable citations can arise from various factors, such as parsing strategies, different object identifiers, crosswalks, etc. Therefore, it is crucial to identify such patterns to ensure the quality of the citation data and produce uCite. The following subsections introduce several main systematic patterns of unreliable citations with examples.

#### 4.3.1 Identity and reference matching issues

Identity issues indicate that paper records are mapped to unreliable PMIDs, such as more than one PMID and incorrect PMIDs. Such issues can occur for various reasons, such as crosswalks from sources to PMIDs, republication of classic articles, book reviews with the same title, different versions of software, tools, and data, and repeat versions of articles and systematic reviews, like Cochrane. For example, the DOIs can be matched into incorrect or multiple PMIDs. One common reason is the duplicated records to which multiple DOIs are assigned. When integrating the citations of these duplicated paper records, citation counts would be miscalculated. Another pattern is due to unreliable reference mapping from PMID to PMCID. For instance, the paper with PMID 24747217 is linked to an incorrect PMCID PMC4991619 record, of which the PMID should be 26579945. This issue will cause the paper to contain 224 cited papers with 110 unreliable ones. As a result, citation sources that use PMC as a source will inherit this unreliable list of cited papers, like iCite and Lens. We remap the duplicated records with multiple PMIDs to one to tackle identity issues. Furthermore, reference matching issues refer to unreliable citation pairs; either citing or cited PMID is unreliable. Reference matching issues can be inherited from identity issues or caused by metadata ambiguity, etc.

#### 4.3.2 Error analysis

Error analyses are conducted to understand the systematic patterns of identity and reference matching issues. This section analyzes three significant causes: ambiguous metadata, typographical errors/variations, and multi-article documents.

**Ambiguous metadata.** Sources could hardly capture papers with ambiguous metadata, like titles, journal names, volume, issue, page numbers, etc. Moreover, metadata ambiguity leads to similar reference strings, which matching procedures can be difficult to distinguish.

Titles containing numbers, chemical formulas, or special characters are ambiguous for matching systems. One concrete example is that the title of one paper with PMID 7328536 is “2010,” of which PubMed Central does not capture the complete list of cited papers. Another example is the papers with highly similar titles, such as “*Cognitive Impairment Following Traumatic Brain Injury*.” vs. “*Cognitive impairment following traumatic brain injury*: the effect of pre- and post-injury administration of scopolamine and MK-801.”

The AJVN ambiguity error refers to a case where the author name, journal name, volume, and issue number are shared by different articles, as illustrated in Fig. 1 on the right panel. The unreliable citation pair, 1 and 4972129, is identified because the unreliable citation shares similar author names, journal names, volume, and issue numbers as the reliable one (PMID 4972128), e.g., “*A B Makar, G J Mannering, Mol Pharmacol. 1968 Sep;4(5):484-91*.” vs. “*A B Makar, T R Tephly, G J Mannering, Mol Pharmacol. 1968 Sep;4(5):471-83*.”

Journal, venue, volume, and page (JVP) errors frequently occur since some matching procedures may weigh only one or some aspects of JVP. Some journals repeat the volume number, e.g., Lancet reused volumes I and II across many years (before 1994), and as a result, volume 1, page 50 could match 20 different papers. Sometimes, journal names are ambiguous for matching systems, for example, journal vs. sub-journal names. The journal Nature was split into Nature Physical Sciences, Nature New Biology, and Nature from 1971 to 1974. The journal names “Nature” and “Nature New Biology” become ambiguous for matching systems and lead to ambiguous reference strings that are difficult to distinguish. “*Nat New Biol. 1972 Jul 12;238(80):37-41. doi: 10.1038/newbio238037a0*.” and “*Nature. 1972 Jul 7;238(5358):37-8. doi: 10.1038/238037a0*.” are two reference strings of two different papers, 12635268 and 18663849. With similar journal names and DOIs, the same year, month, issue number, and first-page number, PubMed Central misidentified the former one, 12635268, as one cited by paper 1917878. Additionally, highly similar reference strings can be caused by similar page numbers and DOIs, e.g., “*Nature. 1970 Aug 15;227(5259):680-5. doi: 10.1038/227680a0*.” and “*Nature. 1970 Jul 4;227(5253):68-9. doi: 10.1038/227068a0*.”. As a result, PubMed Central mismatches the latter as the cited paper of the paper PMID 1714514.

**Typographical errors/variations**. Typographical errors or variations indicate the errors or variations that happen in the metadata, such as a wrong page number corresponding to another article or extreme ambiguity of the title. Typological errors or variations can lead to incorrect matches predicted by the matching system. As shown in Fig. 1, PMID 4754856 is identified as an unreliable citation because the volume number (22) is incorrectly written as 21 in the reference string by the paper author. The reference string “*B. Tabakoff and W. O. Boggan, J. Neurochem. 21, 759*” should be “*B. Tabakoff and W. O. Boggan, J. Neurochem. 22, 759*”. Additionally, the title with chemical formula might be parsed incorrectly, e.g., the title of the paper with PMID 10508365 is partially identified incorrectly as “*In*” vs. “[*In*3(In2)3(PhP)4(Ph2P2)3Cl7(PEt3)3] – A New Molecular III/V Compound Featuring an Unusual 19-Atom Cage”. JVP errors can be caused by typographical errors/variations. For instance, duplicated records are generated by a typographical error in the issue numbers, such as “Nature. 1971 Oct 13;233(*5320*):199-203.” vs. “Nat New Biol. 1971 Oct 13;233(*41*):199-203.”. Journal Nature New Biology volume 233 doesn't contain issue 5320 but issue 41. Consequently, one paper is assigned to two PMIDs.

**Multi-paper documents.** Some journals or issues contain multiple papers on the same page. This will lead to ambiguity about which papers the reference string belongs to. For example, Lancet contains half-page papers on the Lancet, October 28, 1978, pp.915. PDF articles may contain multiple articles or hidden text unrelated to the article. Some publishers include the list of reference stings and the list of citing papers when parsing from PDF.

### 4.4 Inflated citation counts

We further analyze the difference in unreliable citation counts from different sources via the inflated cited paper counts and inflated citing paper counts. Here, compared to the true citation count, inflated cited paper counts refer to an unreliablely high number of citation pairs assigned to a cited paper by a particular source. The inflated citing paper counts refer to an unreliablely high number of citation pairs assigned to a citing paper, compared to the true citation count.

#### 4.4.1 Inflated cited counts

Table 4 samples papers with the most inflated cited counts from each of the nine open-access sources, and compares the total cited counts and unreliable cited counts across uCite, Google Scholar, Scopus, Web of Science, and nine open-access citation sources. The inflation of the cited counts may be due toambiguity, out-of-scope cited papers, and duplicated publications. For example, PMID 19622551 is a case of duplicate publication, where Ann Intern Med (PMID 19622511) is the primary publication venue, and the secondary publications include PMIDs 19631508 (J Clin Epidemiol), 19622551 (BMJ), 19621072 (PLoS Med), and 21603045 (Open Med). The publisher's website for PMID 19622511 (Ann Intern Med) says the following:

**Table 4:** Cited counts for a sample of the cited papers with the most inflated counts. The topmost unreliable count per source is highlighted in bold. Cited counts are separated by Total (T) and Unreliable (U). GS = Google Scholar records linked to corresponding PMID as of 12/21/2024. GS counts are generally higher than uCite because GS includes citing papers that are out-of-scope (i.e., more recent than 2018 or outside PubMed.) and, in some cases, * counts are from multiple PMIDs (for {14974761,15661684} and {19622551,19622511,19631508,19621072,21603045}).

	PMID	uCite	GS	ICE	PMC	OCI	MAG	AMI	SEM	LEN	PAT	DIM	SCO	WOS
T	11422885	75	403	**1871**	**1360**	60	22	0	31	1690	11	1912	43	36
U	11422885			**1813**	**1342**	0	1	0	0	1619	0	1845	0	0
T	5637478	236	277	1018	218	**2055**	1281	0	6	2456	1	2600	4	19
U	5637478			788	0	**2040**	1271	0	0	2221	0	2368	0	0
T	5116655	9	24	5	2	15	**61852**	1	0	8	0	6	2	11
U	5116655			0	0	9	**61851**	0	0	0	0	0	0	0
T	14974761	3068	14369*	2606	537	2545	2238	**7826**	503	3072	243	2885	2850	2328
U	14974761			2	0	2	193	**6938**	21	184	0	5	1	0
T	19622551	5437	133447*	5326	1987	5245	7011	3376	**17975**	5608	1644	5867	1050	783
U	19622551			9	1	404	5407	2551	**14671**	385	0	498	0	0
T	14247882	0	0	0	0	0	9752	0	0	**14122**	0	0	1	0
U	14247882			0	0	0	9752	0	0	**14122**	0	0	0	0
T	29692469	94	2122	85	49	64	0	0	27	207	**72**	145	0	0
U	29692469			0	0	0	0	0	3	114	**26**	51	0	0
T	6110810	225	543	209	79	198	104	87	131	225	44	**27708**	106	106
U	6110810			1	0	1	1	0	1	1	0	**27485**	0	0

*“Editor's Note: In order to encourage dissemination of the PRISMA Statement, this article is freely accessible on the Annals of Internal Medicine Web site (**www.annals.org**) and will be also published in PLOS Medicine, BMJ, Journal of Clinical Epidemiology, and Open Medicine*.”

The paper also appears in PubMed as a reprint in Phys Ther (PMID 19723669), and an accompanying paper with a very similar but longer title is also duplicated across venues: PMIDs 19622512 (Ann Intern Med), 19621070 (PLoS Med), 19622552 (BMJ), 19631507 (J Clin Epidemiol). Furthermore, the PRISMA statement has been updated and published in the years after 2009. This case illustrates that citation analysis, such as distributing citation credit to the authors and journals, can be highly problematic for duplicate publications and their versions, especially without a uniform definition of paper identity across sources.

We further list the metadata of the sample of cited papers with the most inflated cited counts (the top paper for each source).

**ICE** and **PMC** 11422885: Hori T, Sugita Y, Koga E, Shirakawa S, Inoue K, Uchida S, Kuwahara H, Kousaka M, Kobayashi T, Tsuji Y, Terashima M. Proposed supplements and amendments to ‘A manual of standardized terminology, techniques and scoring system for sleep stages of human subjects’, the Rechtschaffen & Kales (1968) standard. Psychiatry & Clinical Neurosciences. 2001; 55(3), 305-310.

**OCI**5637478: Mogensen CE. The glomerular permeability determined by dextran clearance using Sephadex gel filtration. Scandinavian journal of clinical and laboratory investigation. 1968; 21(1): 77-82.

**MAG**5116655: Hirata F, Nakazawa A, Nozaki M, Hayaishi O. Studies on Metapyrocatechase: IV. CIRCULAR DICHROISM AND OPTICAL ROTATORY DISPERSION. Journal of Biological Chemistry. 1971; 246(19): 5882-7.

**AMI**14974761: Jemal A, Tiwari RC, Murray T, Ghafoor A, Samuels A, Ward E, Feuer EJ, Thun MJ. Cancer statistics, 2004. CA: a cancer journal for clinicians. 2004; 54(1): 8-29.

**SEM**19622551: Moher D, Liberati A, Tetzlaff J, Altman DG, PRISMA Group. Preferred reporting items for systematic reviews and meta-analyses: the PRISMA statement. BMJ. 2009; 339: b2535.

**LEN**14247882: ROWLAND M, TURNER P. AKUFO AND IBARAPA. Lancet (London, England). 1965; 1(7380): 307-8.

**PAT**29692469: Sikes RS, Animal Care and Use Committee of the American Society of Mammalogists. 2016 Guidelines of the American Society of Mammalogists for the use of wild mammals in research and education. Journal of mammalogy. 2016; 97(3): 663-88.

**DIM**6110810: Paxinos G, Watson CR, Emson PC. AChE-stained horizontal sections of the rat brain in stereotaxic coordinates. Journal of neuroscience methods. 1980; 3(2): 129-49.

#### 4.4.2 Inflated citing counts

Table 5 summarizes the most inflated citing papers in the nine open-access sources, plus Scopus and Web of Science. For example, ICE identifies that paper PMID 22386883 cites 1645 papers, of which 933 are confirmed unreliable. A sample of citing papers with the most inflated citing counts (the top paper for each source):

**Table 5:** Citing counts for a sample of the citing papers with the most inflated counts. The topmost unreliable count per source is highlighted in bold. Citing counts are separated by Total (T) and Unreliable (U). # Some, not all, of the papers cited by PMID 15495084 and identified by OCI and DIM are incorrect because they are sourced from an updated version the paper, PMID 18677777, with an overlapping set of cited papers. * One source of the cited papers, PMCID 5583682, incorrectly maps to PMID 27378485; the uCite dataset has been corrected so that the PAT citing count is 0 but it would have been 122 without the correction.

	PMID	uCite	ICE	PMC	OCI	MAG	AMI	SEM	LEN	PAT	DIM	SCO	WOS
T	22386883	713	**1645**	**1379**	699	0	0	665	1413	0	1645	0	683
U			**933**	**932**	0	0	0	16	700	0	933	0	0
T	15495084	0	0	0	**339**	0	0	0	0	0	410	0	0
U			0	0	**339#**	0	0	0	0	0	410#	0	0
T	17731104	0	0	0	0	**726114**	0	0	0	0	0	0	0
U			0	0	0	**726114**	0	0	0	0	0	0	0
T	15039941	223	0	0	0	0	**9916**	0	0	0	223	0	0
U			0	0	0	0	**9869**	0	0	0	0	0	0
T	29986069	60	60	60	0	0	0	**5343**	60	60	60	0	0
U			0	0	0	0	0	**5342**	0	0	0	0	0
T	22058406	82	81	81	77	6324	4476	1555	**8219**	82	81	78	75
U			0	0	0	6243	4417	1546	**8137**	0	0	0	0
T	27378485	86	210	122	86	0	0	0	209	122*	211	0	0
U			124	122	0	0	0	0	123	122*	125	0	0
T	15687843	0	0	0	0	0	0	0	0	0	**1454**	0	0
U			0	0	0	0	0	0	0	0	**1454**	0	0

**ICE and PMC**22386883: Calkins H, Kuck KH, Cappato R, Brugada J, Camm AJ, Chen SA, Crijns HJ, Damiano Jr RJ, Davies DW, DiMarco J, Edgerton J. 2012 HRS/EHRA/ECAS expert consensus statement on catheter and surgical ablation of atrial fibrillation: recommendations for patient selection, procedural techniques, patient management and follow-up, definitions, endpoints, and research trial design: a report of the Heart Rhythm Society (HRS) Task Force on Catheter and Surgical Ablation of Atrial Fibrillation. Developed in partnership with the European Heart Rhythm Association (EHRA), a registered branch of the European Society of Cardiology (ESC) and .... Europace. 2012; 14(4): 528-606.

**OCI**15495084: Bjelakovic G, Nikolova D, Simonetti RG, Gluud C. Antioxidant supplements for preventing gastrointestinal cancers. Cochrane Database Syst Rev. 2004 Oct 18:(4):CD004183. An updated version was published in 2008 (PMID 18677777).

**MAG**17731104: Wilson EB. Proceedings of the National Academy of Sciences. Science. 1917; 45(1159): 261-3.

**AMI**15039941: ***No authors listed***. Current literature in mass spectrometry. J Mass Spectrom. 2004; 39(3): 329-40.

**SEM**29986069: Deshpande AP, Collins K. Mechanisms of template handling and pseudoknot folding in human telomerase and their manipulation to expand the sequence repertoire of processive repeat synthesis. Nucleic acids research. 2018; 46(15): 7886-901.

**LEN**22058406: Greco M, Chiappetta A, Bruno L, Bitonti MB. In Posidonia oceanica cadmium induces changes in DNA methylation and chromatin patterning. Journal of experimental botany. 2012; 63(2): 695-709.

**PAT**27378485: Mashaghi A, Hong J, Chauhan SK, Dana R. Ageing and ocular surface immunity. British Journal of Ophthalmology. 2017;101(1):1-5.

**DIM**15687843: ***No authors listed***. Bibliography. Current world literature. Clinical nephrology. Curr Opin Nephrol Hypertens. 2005; 14(2): 161-79.

## Limitations

uCite prioritizes citations with PubMed Identifiers (PMIDs) before 2018. Consequently, citations beyond this scope, which do not possess PMIDs, are not integrated into our dataset. To ensure the inclusion of citations from diverse sources lacking PMIDs, we have implemented several parsing techniques and established strategies to assess the reliability of the parsed citations. Nevertheless, there remains a slight possibility that reliable citations may be misclassified as unreliable during this process. This potential issue arises when citations are covered by a single or a limited number of sources.

## Ethics Statement

Authors have read and followed the ethical requirements for publication in Data in Brief, confirming that the current work does not involve human subjects, animal experiments, or data collected from social media platforms.

## CRediT authorship contribution statement

**Liri Fang:** Writing – original draft, Writing – review & editing, Visualization. **Malik Oyewale Salami:** Writing – review & editing. **Griffin M. Weber:** Resources, Writing – review & editing. **Vetle I. Torvik:** Conceptualization, Methodology, Data curation, Visualization, Writing – original draft, Writing – review & editing.

## Data Availability

Illinois Data BankuCite: The union of nine large-scale public PubMed citation datasets with reliability filtering (Original data).
